# When a skull base paraganglioma reaches the heart

**DOI:** 10.1093/ehjimp/qyag091

**Published:** 2026-05-21

**Authors:** Małgorzata Wojciechowska, Dominik Wretowski, Krzysztof Kądziołka, Zuzanna Zalewska, Kinga Kowalik, Maciej Zarębiński

**Affiliations:** Chair and Department of Experimental and Clinical Physiology, Laboratory of Centre for Preclinical Research, Medical University of Warsaw, Banacha 1B, Warsaw 02-097, Poland; Neurosurgery Department, Independent Public Specialist Western Hospital John Paul II, Grodzisk 05-825 Mazowiecki, Poland; Neurosurgery Department, Independent Public Specialist Western Hospital John Paul II, Grodzisk 05-825 Mazowiecki, Poland; Chair and Department of Experimental and Clinical Physiology, Laboratory of Centre for Preclinical Research, Medical University of Warsaw, Banacha 1B, Warsaw 02-097, Poland; Chair and Department of Experimental and Clinical Physiology, Laboratory of Centre for Preclinical Research, Medical University of Warsaw, Banacha 1B, Warsaw 02-097, Poland; Department of Invasive Cardiology, Independent Public Specialist Western Hospital John Paul II, Lazarski University, Grodzisk Mazowiecki 05-825, Poland

A 44-year-old woman presented with severe, treatment-resistant headaches, progressive right-sided hearing loss, and mild fatigue. She had no other neurological or cardiological symptoms, and her blood pressure was normal.

Head and neck MRI revealed a highly vascular lesion arising in the jugulo-tympanic region with a characteristic ‘salt-and-pepper’ appearance, infiltrating the middle ear and extending into the skull base venous sinuses (*Figure 1A–C*). Intraluminal tumour spread via the right internal jugular vein and superior vena cava into the right atrium was confirmed by chest CT and transthoracic echocardiography (*Figure 1D, E*).

**Figure 1:**
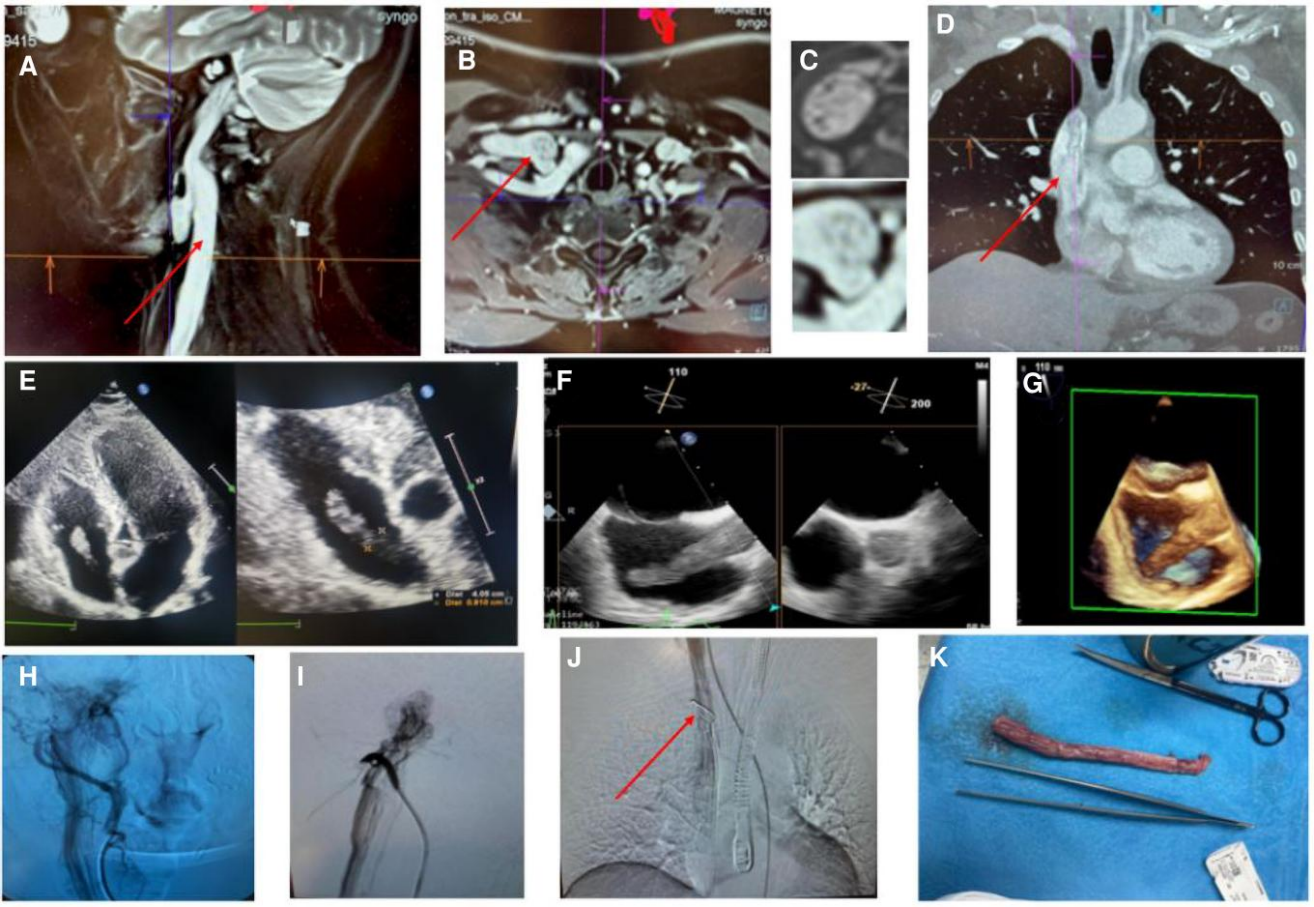
(*A*) MRI of the head and neck, the tumour spreading from the jugulotympanic region down via venous vascular system (arrow). (*B*) Axial MRI of the venous angle, where the internal jugular vein joins the subclavian vein, showing a tumour with a characteristic ‘salt-and-pepper’ appearance (arrow). (*C*) Magnified MRI view showing the ‘salt-and-pepper’ sign, reflecting flow voids (‘pepper’) and hyperintense tumour tissue (‘salt’), typically seen in highly vascular tumours. (*D*) Chest CT showing the tumour extending from superior vena cava into the right atrium (arrow). (*E*) TTE, apical view, showing a tumour in the right atrium reaching the level of the tricuspid valve. Echocardiographic assessment showed a normal right heart with no TR, no RA dilatation and no pulmonary hypertension. The endocardial portion of the mass measures approximately 4 cm in length and almost 1 cm in diameter. (*F*) TEE 2D: the tumour protruding from superior vena cava to the right atrium in bicaval view (on the left); superior vena cava in transverse section completely occupied by the tumour (on the right). (*G*) TEE: A finger-like tumour on 3D imaging. (*H*) Non-selective angiography of the right external carotid artery showing pathological tumour vascularization. (*I*) Selective angiography of the right occipital artery arising from the right external carotid artery showing pathological tumour vascularization. (*J*) TEE-guided biopsy performed during the angiography procedure. The mass was captured with a vascular loop, and a fragment was aspirated using the 12F Penumbra Indigo Lightning system (biopsy loop indicated by an arrow). Both angiography and embolization procedures were performed via transradial access (TRA, 7F). (*K*) Macroscopic view of the resected tumour. A venotomy of the internal jugular vein was performed. The tumour, hanging loosely, was extracted from the superior vena cava and right atrium. The upper portion of the mass was mobilized with gentle traction and then resected using electrocautery. The excised tumour measured approximately 18 cm in length.

Transoesophageal echocardiography (TEE) revealed a finger-like mass within the right atrium, hanging freely from the superior vena cava up to the level of the tricuspid valve, without infiltration of surrounding tissues (*Figure 1F, G*).

Due to the tumour location, surgical resection was initially considered high-risk; angiography was performed to map the vascular anatomy (*Figure 1H, I*). A TEE-guided biopsy was obtained to determine whether the intracardiac component represented tumour or thrombus, confirming paraganglioma (*Figure 1J*).

Two embolization sessions were necessary to achieve sufficient tumour devascularization. Complete surgical resection was then performed via a cervical approach (*Figure 1* K).

Rivaroxaban 20 mg was administered from the time of diagnosis of venous involvement by the tumour until definitive therapy.

At two-year follow-up, the patient remains clinically stable with no evidence of recurrence, although right-ear hearing loss persists.

Skull base paragangliomas are rare, highly vascular neoplasms that most commonly arise from the paraganglionic tissue associated with glossopharyngeal (IX) or vagus (X) nerves. They are typically hormonally inactive. Management is individualized and may include embolization, surgical resection, and, in selected cases, radiotherapy.

## Lead author biography


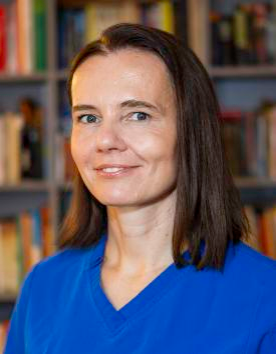
I graduated in 2002 and completed specialist training in internal medicine and cardiology, followed by a PhD focused on myocardial ischaemia–reperfusion injury and cardioprotective conditioning strategies. I work as a hospital cardiologist and researcher, and I am also an award-winning academic teacher. My main interests include acute cardiovascular care and echocardiography, with particular emphasis on intra-procedural imaging.

